# Mechanical Bowel Obstruction Due to Secondary Sclerosing Peritonitis, Managed Conservatively: A Case Report

**DOI:** 10.7759/cureus.51683

**Published:** 2024-01-05

**Authors:** Laith Daraghmeh, Malak Eleiwi, Naim Qamhia, Iyad Maqboul

**Affiliations:** 1 General Surgery, An-Najah National University Hospital, Nablus, PSE; 2 Medicine, An-Najah National University Hospital, Nablus, PSE; 3 Pathology, An-Najah National University Hospital, Nablus, PSE

**Keywords:** conservative and surgical treatment, peritoneal dialysis (pd), surgical case reports, small intestinal obstruction, sclerosing peritonitis

## Abstract

An uncommon cause of intestinal obstruction is an abdominal cocoon, also known as sclerosing encapsulating peritonitis (SEP). We present the case of a 24-year-old female peritoneal dialysis patient who presented with a picture of complete intestinal obstruction. After reviewing the patient's medical history and acquiring relevant laboratory and imaging data, the decision was made to proceed with surgery. Intraoperatively, however, she had a picture of sclerosing peritonitis. The decision was to terminate the surgery and to take a conservative approach, including total parenteral nutrition. Her condition improved, obstruction was resolved, and she was discharged home in good clinical condition. Sclerosing peritonitis should be considered a possible etiology that can be managed conservatively in any peritoneal dialysis patient with intestinal obstruction.

## Introduction

Sclerosing encapsulating peritonitis (SEP) is an uncommon form of peritoneal inflammation that can be fatal. Sclerosing peritonitis is usually linked with peritoneal dialysis; however, it can also develop after renal or liver transplantation or have associations with particular medication regimens [1]. It has been classified as either idiopathic or secondary. Foo et al. identified the idiopathic type, also known as abdominal cocoon, in 1978. Intestinal obstruction caused by an abdominal cocoon is relatively rare [2]. Peritoneal dialysis is one of the main hypothesized causes of SEP. The length of time on peritoneal dialysis is an important factor, especially for people who have been on dialysis for more than four to five years [3].

Many therapeutic options for the secondary form of this condition have been proposed, including: abandoning peritoneal dialysis and shifting to hemodialysis; nutritional support; steroids and tamoxifen; and surgery for intestinal obstruction [2]. Due to the low incidence of sclerosing peritonitis, there is no expert agreement on whether the treatment of choice should be surgical or conservative; according to a 2011 paper, no respective clinical trials have compared the different therapeutic options, and experience is confined to case reports [1]. However, according to a 2016 review article, individuals with minor abdominal symptoms should be managed conservatively with bowel rest, nasogastric decompression, and either enteral or parenteral nutrition [4]. We describe a successful conservative approach that includes canceling surgery as soon as the diagnosis is suspected and, subsequently, initiating inpatient total parenteral nutrition.

## Case presentation

A 24-year-old female patient with Down syndrome and cerebral palsy was admitted to our surgical ward from the emergency department after presenting with paroxysmal attacks of abdominal pain for nearly a year. Her chronic pain was generalized and colicky and increased by eating, with accompanying weight loss and diarrhea.

Her medical history was notable for the following: vesicourethral reflux since early life; and end-stage renal disease. She began regular dialysis three years prior to presentation, initially using a peritoneal dialysis catheter for roughly two and a half years, with the catheter later removed due to malfunction. She was thereafter on regular hemodialysis via a left brachiocephalic shunt.

Many investigations were carried out after her family repeatedly sought medical advice, but no definitive diagnosis was determined, and she was treated conservatively. She eventually got a diagnosis of familial Mediterranean fever (FMF), which was supported by genetic testing, given that FMF runs in her family. After being prescribed colchicine, her symptoms did not improve. She presented to the emergency department because she had been experiencing more pain and recurrent bilious vomiting for the last several days, which was interfering with her daily tasks. Her parents mentioned that they had not sought medical advice before as they thought that her symptoms were due to FMF exacerbations.

Her vitals on admission included a heart rate of 102 beats per minute, a BP of about 100/50 mmHg, and a temperature of 36.9 axillary. The physical examination revealed significant abdominal distention, entire abdominal tenderness with guarding, and no active bowel sounds. Routine laboratory testing indicated a white blood cell count of 10.75 k/µl, platelets of 252 k/µL, hemoglobin of 7.3 g/dL, and CRP of 39.6 mg/L. Table 1 shows initial laboratory results upon presentation.

**Table 1:** Laboratory results upon admission BUN: Blood urea nitrogen, CRP: C-reactive protein

Parameter	Result	Reference range
White blood cell count	10.75	4 - 9 k/µl
Neutrophil %	83	50 % - 70 %
Lymphocyte %	10.4	18 % - 42 %
Hemoglobin	7.3	13.7 - 17.2 g/dL
Platelets	252	140 - 450 k/µl
BUN	6.11	5 - 22 mg/dL
Creatinine	2.52	0.7 - 1.2 mg/dL
Magnesium, serum	2.06	1.6 - 2.6 mg/dL
Phosphate, serum	1.9	2.5 - 4.5 mg/dL
Sodium	135	135 - 155 mEq/L
Potassium	3	3.5 - 4.8 mEq/L
Chloride	89.2	98 - 107 mEq/L
Prothrombin time	12.3	11 - 14 Sec
International normalized ratio	0.94	0.8 - 1.2
Activated partial thromboplastin time	29.4	25 - 40 Sec
CRP (quantitative)	39.6	0 - 5 mg/L

**Figure 1:**
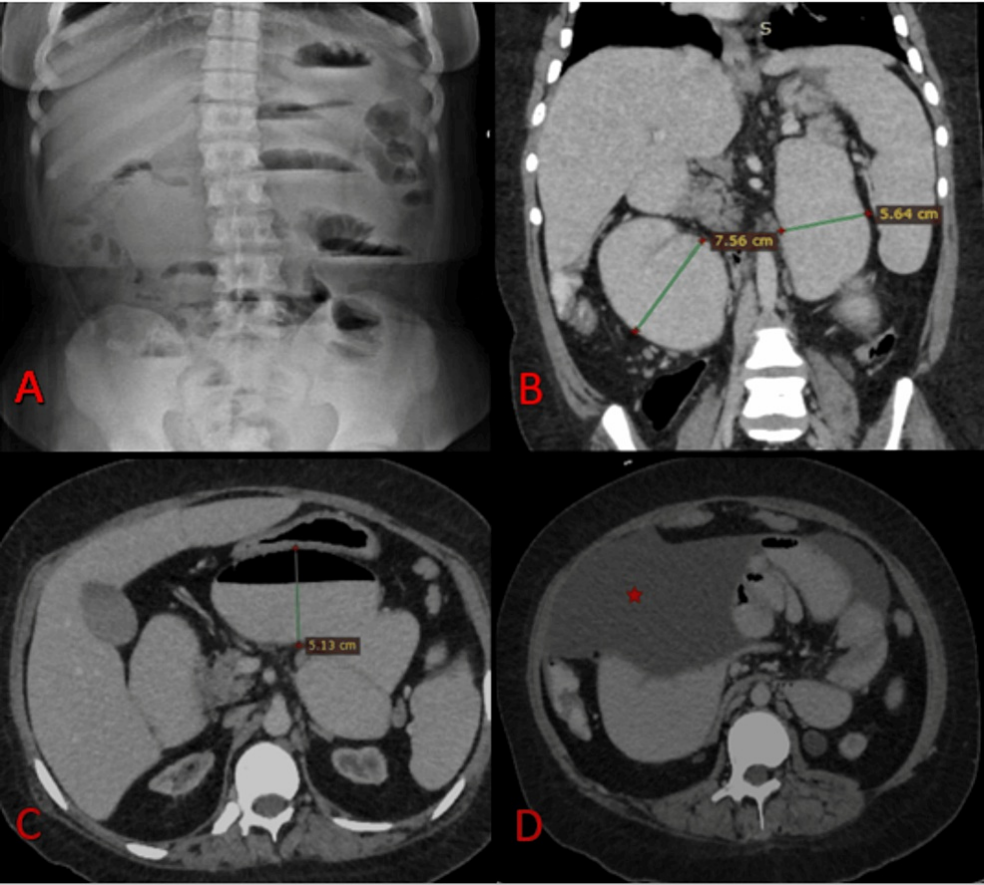
Imaging of the patient at the time of admission A: A standing abdomen x-ray reveals multiple air-fluid levels, indicating an intestinal obstruction; B and C: CT of the abdomen, showing dilated small bowel loops in the coronal and axial sections, respectively; D: Axial view of the abdomen CT reveals free fluid in the abdomen (red star)

A CT scan of the abdomen with IV and oral contrast revealed upper small bowel obstruction with gross dilatation of the duodenum and upper jejunum measuring up to 6 cm in diameter (Figure 1).

**Figure 2:**
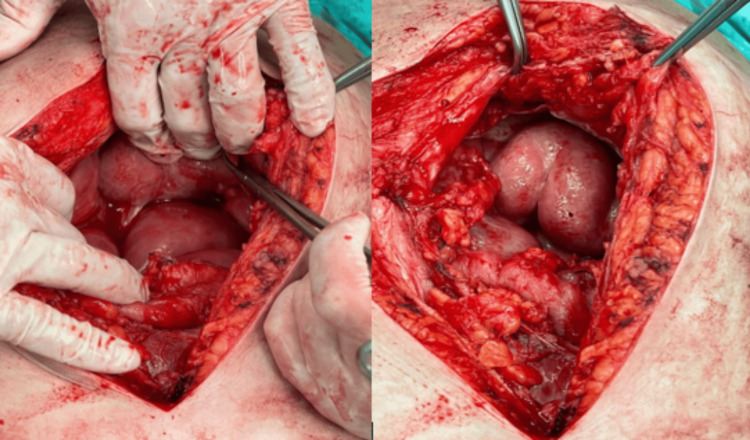
Intraoperative findings show a thick fibrous coat on the small bowel that is suggestive of sclerosing peritonitis.

An exploratory laparotomy was done. Intraoperatively, the abdominal cavity was frozen, making bowel mobilization and adhesiolysis impossible without significant bowel injuries (Figure 2).

**Figure 3:**
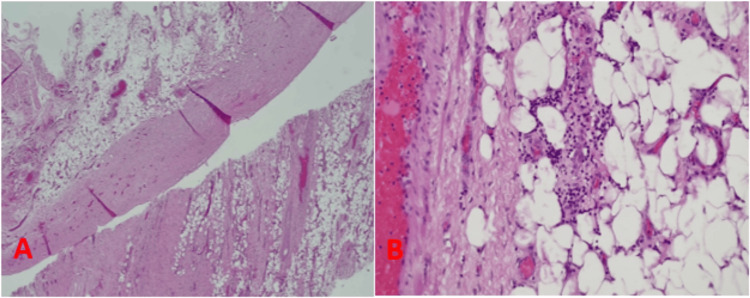
Histopathologic examination The tissue showed dense collagenous fibrous bands, mostly at the surface of the omental tissue. In some areas, it appeared that the fibrosis extended deeper into fatty tissue, as seen at the bottom right part of Figure A. Apparent denudation of the surface mesothelium and prominent vascularity in affected areas were noted. Inflammation was not the predominant picture, but there were very small foci of inflammation, predominated by chronic inflammatory cells. A: Thick collagenous fibrous bands are seen covering omental tissue with denuded mesothelial lining (H&E stain at 20x); B: Small foci of inflammation predominated by small lymphocytes (H&E at 200x) H&E: Hematoxylin and eosin

There was a serous fluid collection of about 900 ml, surrounded by a nodular, thick wall. The fluid was evacuated and sent for cytology, which revealed a few scattered macrophages and no malignant cells, and part of the wall was sent for histopathologic examination (Figure 3). Given the likelihood that these findings would demonstrate sclerosing peritonitis, the decision was made to stop the surgery and adopt a conservative strategy that included total parenteral nutrition to manage the obstruction. Therefore, a central line was inserted.

The patient was transported to the ward postoperatively to complete her care. She was closely monitored by the nephrology staff, who kept a tight intake and output chart and evaluated her regularly. She maintained nil per os with total parenteral nutrition and IV fluids after the nephrology and pharmacology specialist teams evaluated her. She was followed every two to three days with an abdomen X-ray that showed no worsening but improvement.

**Figure 4:**
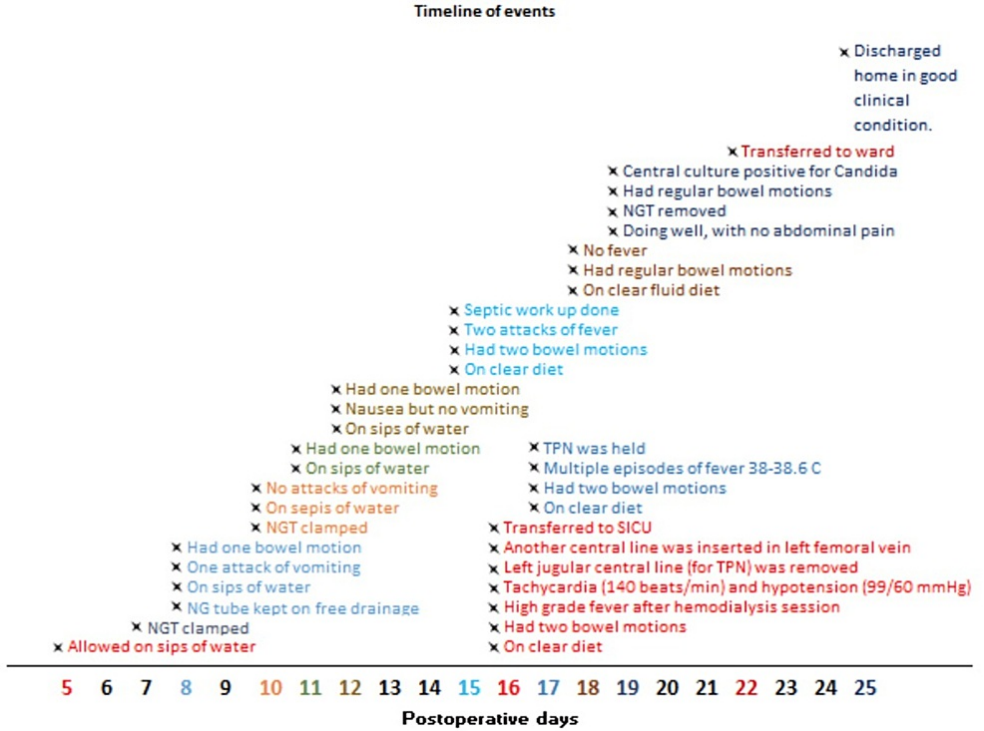
Patient's course of hospitalisation NGT: Nasogastric tube, TPN: Total parenteral nutrition, SICU: Surgical intensive care unit

Regarding her hospital stay, she maintained nil per os for the first five days after surgery since she was still experiencing abdominal distension and pain with no bowel movements and ongoing bilious output via her nasogastric tube at an amount of approximately 1500 ml per day. She was in the hospital for a total of 25 days following surgery, with the nasogastric tube clamped and declamped regularly based on her food tolerance. The nasogastric tube was withdrawn once she established a tolerable clear fluid diet, regular daily bowel movements, and relief of her distension. She was maintained on a soft diet and discharged home. The patient-hospital course is shown in Figure 4. 

It is worth mentioning that the patient was hospitalized in the critical care unit for a total of seven days on postoperative day 16 due to a fever episode with tachycardia and hypotension. After being evaluated, she suggested having an infected internal jugular central line removed, and she was kept on an antibiotic treatment that was changed based on a positive *Candida albicans* culture. After one week, the patient was assessed in the outpatient clinic; she was doing well, with minimal symptoms of abdominal pain, had regular daily bowel motions, and tolerance of a clear to soft diet. 

## Discussion

Sclerosing peritonitis is a rare form of peritoneal inflammation that involves both the visceral and the parietal surfaces of the abdominal cavity. A fibrous thickening of the peritoneum characterizes sclerosing peritonitis. Sclerosing encapsulating peritonitis can be described as primary (idiopathic) or secondary. Primary SEP, also known as abdominal cocoon syndrome, is divided into three categories based on the degree of encasement by the membrane [4]. Primary SEP is idiopathic, meaning it has no known etiology.

Secondary SEP occurs more frequently than idiopathic SEP. The most prevalent cause of secondary SEP is peritoneal dialysis. Several studies have found a link between prolonged peritoneal dialysis and the development of secondary SEP. A history of abdominal surgery, autoimmune diseases, certain medicines, peritoneal shunts, and recurring bouts of peritonitis are among the less common causes of secondary SEP [5].

These patients tend to present with recurrent abdominal pain, nausea, vomiting, anorexia, weight loss, and malnutrition, while some may be asymptomatic. Recurrent bouts of non-strangulating obstruction, paired with pertinent imaging abnormalities and a lack of alternative etiologies, may raise a high index of clinical suspicion [2]. A preoperative diagnosis is critical, especially in a peritoneal dialysis patient with a clinical picture of intestinal obstruction with concomitant imaging findings, since this may avert unnecessary procedures for the patient, such as surgery. In our situation, no preoperative diagnosis was made; nonetheless, given intraoperative suspicions of the diagnosis, no surgical intervention such as adhesiolysis or bowel resection was performed.

According to a 2016 review article, individuals with minor abdominal symptoms should be managed conservatively with bowel rest, nasogastric decompression, and either enteral or parenteral nutrition, with a drug therapy that may include tamoxifen, steroids, colchicine, azathioprine, and mycophenolic acid in those who failed to respond to a conservative approach [4]. Despite the initial severe picture of obstruction in this patient, we decided to take a conservative strategy, which justified maintaining her away from the operation theater for a high-mortality adhesiolysis procedure. According to studies, the overall death rate in SP patients was 24%, while the mortality rate in patients who received surgical therapy was 43% [1].

A review of 118 cases showed that the vast majority of patients were diagnosed on the operating table. Almost all of the patients (99.2%) had surgical exploration, which consisted of either membrane excision or adhesiolysis (100%), as well as extra procedures such as resection, anastomosis, mesenteric plication, or intestinal stenting. The results were largely favorable, with just 5.9% of patients experiencing recurring obstructions. One patient received conservative therapy and died as a result of liver failure; this was the only death recorded (0.8%) [4].

While the diagnosis was made intraoperatively in our case, we chose not to perform adhesiolysis or any of the other procedures stated in the review by Machado [4]. Instead, we took the specimen for histopathologic examination and chose a conservative approach.

## Conclusions

Sclerosing peritonitis is a rare cause of intestinal obstruction. A preoperative diagnosis requires a high degree of suspicion. This should make the attending clinician much more cautious, lower the threshold for operative intervention, and use conservative therapy whenever possible to avoid the high mortality associated with surgery, especially for those with end-stage renal disease on peritoneal dialysis.
